# Durability of Humoral Immune Responses to SARS-CoV-2 in Citizens of Ariano Irpino (Campania, Italy): A Longitudinal Observational Study With an 11.5-Month Follow-Up

**DOI:** 10.3389/fpubh.2021.801609

**Published:** 2021-12-17

**Authors:** Annachiara Coppola, Carlo Buonerba, Davide Cardinale, Gabriella Lo Conte, Donato Sansone, Giuseppe Rofrano, Sabato De Vita, Maria Morgante, Maria Triassi, Luigi Atripaldi, Gianfranco Brambilla, Rocco Sabatino, Andrea Pierri, Daniela Pacella, Antonio Pizzolante, Biancamaria Pierri, Veronica Ferrucci, Massimo Zollo, Mario Capasso, Silvia Stringhini, Paolo Antonio Ascierto, Sante Roperto, Pellegrino Cerino

**Affiliations:** ^1^Centro di Referenza Nazionale per l'Analisi e Studio di Correlazione tra Ambiente, Animale e Uomo, Istituto Zooprofilattico Sperimentale del Mezzogiorno, Portici, Italy; ^2^Dipartimento di Medicina Sperimentale, Universita' degli studi della Campania “L. Vanvitelli”, Naples, Italy; ^3^Azienda Sanitaria Locale Avellino, Avellino, Italy; ^4^Department of Public Health, Federico II University of Naples, Naples, Italy; ^5^Cotugno Hospital, AORN Ospedali dei Colli, Naples, Italy; ^6^Istituto Superiore di Sanità, Food Safety, Nutrition, and Veterinary Public Health Department, Rome, Italy; ^7^CEINGE Biotecnologie Avanzate, Naples, Italy; ^8^Division and Department of Primary Care Medicine, Geneva University Hospitals, Geneva, Switzerland; ^9^Unit of Melanoma, Cancer Immunotherapy & Development Therapeutics, Istituto Nazionale Tumori IRCCS Fondazione Pascale, Naples, Italy; ^10^Dipartimento di Medicina Veterinaria e Produzioni Animali, Università di Napoli Federico II, Naples, Italy

**Keywords:** SARS-CoV-2, seroprevalence, antibody response, immunoassay, screening

## Abstract

As of November 17, 2021, SARS-CoV-2 (Severe Acute Respiratory Syndrome CoronaVirus 2), the causative agent of COVID-19 (COronaVIrus Disease 19), has infected ~250 million people worldwide, causing around five million deaths. Titers of anti-SARS-CoV-2 neutralizing antibodies were relatively stable for at least 9 months in a population-based study conducted in Wuhan, China, both in symptomatic and in asymptomatic individuals. In the mass screening campaign conducted in the town of Ariano Irpino (Avellino, Italy) in May, 2020, 5.7% (95% CI: 5.3-6-1) of the 13,444 asymptomatic citizens screened were positive for anti-nucleocapsid antibodies against SARS-CoV-2. Among these, 422 citizens were re-tested for anti SARS-CoV-2 antibodies in January, 2021 and/or in April, 2021 and enrolled in this longitudinal observational study. Median (interquartile range) age of the study cohort was 46 years (29–59), with 47 (11.1%) participants of minor age, while 217 (51.4%) participants were females. There was no evidence of re-infection in any of the subjects included. Presence of anti-nuclear antibodies antibodies (Elecysis, Roche) was reported in 95.7 and 93.7% of evaluable participants in January and April, 2021. Multiple logistic regression analysis used to explore associations between age, sex and seroprevalence showed that adults vs. minors had significantly lower odds of having anti-S1 antibodies (Biorad) both in January, 2021 and in April, 2021. Our findings showed that antibodies remained detectable at least 11.5 months after infection in >90% of never symptomatic cases. Further investigation is required to establish duration of immunity against SARS-CoV-2.

## Introduction

As of November 17, 2021, SARS-CoV-2 (Severe Acute Respiratory Syndrome Corona Virus 2), the causative agent of COVID-19 (Corona Virus Disease 19), has infected ~250 million people worldwide, causing around five million deaths [World health Organization Health Emergency Dashboard, 17 November 2021, 10.59 am]. SARS-CoV-2 high infectivity along with COVID-19 relatively low mortality represent two key determinants of the global pandemic, which persists despite continued efforts of the international community. Although a wealth of epidemiology (1) and immunology (2) data are now available, the exact duration of immunity after recovering from COVID-19 remains to be established (3), and the anecdotal cases of re-infection have been generally attributed to an infection with a genetically distinct virus, rather than to loss of immunity (4, 5). Durable immunity after recovery from symptomatic COVID-19 was reported in a cohort of 188 COVID-19 cases, mostly with mild disease, with ~95% of subjects presenting with SARS-CoV-2 specific antibodies, memory B cells, CD4+ and CD8+ T cells 6 months after the initial infection. Titers of anti-SARS-CoV-2 neutralizing antibodies were relatively stable for at least 9 months in a population-based study conducted in Wuhan, China, both in symptomatic and in asymptomatic individuals (6).

In May, 2020, we conducted a mass SARS-CoV-2 serological screening campaign in the town of Ariano Irpino (Avellino, Italy) (7), a municipality of ~20,000 inhabitants that was locked down by the regional authorities in April, 2020, because of a steep rise in local COVID-19 cases. In the cohort of 13,444 asymptomatic citizens screened, a sero-prevalence of 5.7% (95% CI: 5.3-6-1) was reported, with 101 citizens positive for SARS-CoV-2 RNA on RT-PCR, which corresponds to 13% (95% CI: 11.3–16.4) of seropositive cases and to 0.7% of the entire population screened.

In the retrospective observational study presented here, we reviewed available longitudinal serological findings obtained in the cohort of seropositive asymptomatic Ariano Irpino citizens recruited in May, 2020. Our main objective was to explore the temporal dynamics of antibody response against SARS-CoV-2 in never symptomatic subjects. Data about semi-quantitative assessment of IgG against SARS-CoV-2 nucleocapsid, receptor-binding domain, spike 1, and spike 2 proteins add novelty to our findings.

## Methods

### Study Design

The study presented here was designed to assess the duration of seropositivity against SARS-CoV-2 in asymptomatic individuals. STROBE recommendations (Strengthening the Reporting of Observational Studies in Epidemiology) were followed for this observational cohort study (8). Citizens who were enrolled in the Ariano Irpino Screening Program in May, 2020 (7), realized jointly by the Zoo-Prophylactic Institute of Southern Italy (Portici, Italy), the Local Health Unit (Azienda Sanitaria Locale—ASL—Avellino, Avellino, Italy), the Department of Public Health of University Federico II (Naples, Italy) and Department of Health Services of Azienda Ospedaliera dei Colli-Cotugno and Monaldi Hospital (Naples, Italy) were offered to be re-tested for anti-SARS-CoV-2 antibodies at various times in facilities located in the town of Ariano Irpino. Demographic and serological findings were recorded in an anonymized database, which we analyzed to conduct this retrospective study. We included participants who met the following two conditions: (1) they were seropositive in May, 2020; (2) they had been re-tested for SARS-CoV-2 antibodies afterwards.

The retrospective observation period started on the first day of the Ariano Irpino Screening Campaign in May, 2020 and lasted for 1 year. All available data regarding age, sex and antibody test results for SARS-CoV-2 were retrieved. The primary objective of the study was to assess duration of seropositivity for anti-SARS-CoV-2 antibodies in asymptomatic individuals. The secondary objective was to assess whether gender (males vs. females) and age (minors vs. adults) may affect seropositivity rates and levels of antibody titers (if available).

### Analytical Tests

#### Qualitative Assay Total Antibodies (IgA-IgM-IgG)

Qualitative assessment of anti-N antibodies (Roche) was carried out at the Laboratory of Microbiology and Virology of the Monaldi Hospital by Monaldi personnel. Antibodies against SARS-CoV-2 were qualitatively assessed in peripheral blood using the anti-SARS-CoV-2 Elecsys E2G 300 assay (Roche Diagnostics). The Elecsys anti-SARS-CoV-2 assay (Roche Diagnostics) is an electrochemiluminescence immunoassay that allows the *in vitro* qualitative detection of antibodies (including IgG) against SARS-CoV-2 in human serum and plasma. This test employs a sandwich reaction that includes both biotinylated and ruthenylated SARS-CoV-2 recombinant nucleocapsid antigens incubated with the sample. The adding of streptavidin-coated microparticles allows the complex to be captured magnetically after binding to the solid phase through a biotin–streptavidin reaction. Electrochemiluminescence emission signals are interpolated to generate test results. Testing requires 12 μl of the sample, and the duration of the procedure is 8 min (9). For diagnostic purposes, the Elecsys anti-SARS-CoV-2 immunoassay (Roche Diagnostics) was performed according to the manufacturer's instructions, and assay results were interpreted as follows: cutoff index <1.0, non-reactive/negative for anti-SARS-CoV-2 antibodies; cutoff index ≥1.0, reactive/positive for anti-SARS-CoV-2 antibodies. Blood samples were centrifugated at 2109 xg for 10 min and aliquots of serum were sent immediately to the laboratory of the Monaldi Hospital where they were analyzed. Samples were kept at 4°C controlled temperature during transportation.

#### Qualitative and Semi-quantitative Assay (IgG Against RBD, Spike1, Spike2, N)

Semi-quantitative assessment of anti-S1, -S2, -N, -RBD antibodies (Biorad) was conducted at the Laboratory of Virology and Microbiology of the public hospital of Catanzaro “Pugliese Ciaccio” by IZSM personnel. Blood samples were centrifugated at 2109 xg for 10 min and aliquots of serum were frozen at −80°C. The day after the collection of samples, aliquots of serum were carried in dry-ice at −80°C to the laboratory of the “Pugliese-Ciaccio” Hospital in Catanzaro, where IZSM researchers carried out the analysis.

Assessment of IgG against SARS-CoV-2 S1, S2, RBD and N antigens in human serum was carried out using the BioPlex 2200 SARS-CoV-2 IgG kit, which employs magnetic microspheres coated with RBD, Spike 1 protein (S1), Spike 2 protein (S2) or nucleocapsid (N) antigens. The analysis was conducted as per manufacture's instructions, as others have done (10, 11), and the laboratory system was calibrated with a set of six vials, to which antigen-specific antibodies at five different concentrations were transferred in order to stabilize the calibration curve.

The results of the test were reported for antigen-specific IgG as follows: cutoff index <9 U/ml, negative for anti-Sars-CoV-2 IgG; cutoff index >10 U/ml, positive for anti-SARS-Cov-2 IgG. Quantitative results ranged between 1 and 100 U/ml. Samples showing results above the assay's limit of quantitation were not further diluted for exact quantitation.

### Statistical Analysis

Median and interquartile range were used to describe the quantitative distribution of antibody among subjects who had between 1 and 100 IU/ml for each essay. Estimates of seroprevalence were computed dividing the raw frequency of positive subjects by the sample size of each group. 95 % confidence intervals for prevalence were estimated using binomial exact method. Differences in prevalence and antibody titles between essays was computed using chi-square or Fisher's exact test as appropriate. Difference in antibody distributions within subjects across essays was compared with one-way repeated measures ANOVA or Friedman's test as appropriate. Difference in antibody distributions across age and sex subgroups was compared with Student's t or Mann-Whitney U test as appropriate. Multiple logistic regression model was used to investigate the association of age and sex with seroprevalence for each of the essays. Model goodness-of-fit was assessed with Akaike Information Criterion (AIC); possible association between the two independent variables age and sex were investigated with Student's *t*-test. For all analyses, a *p* < 0.05 was considered statistically significant. All analyses were conducted using R version 4.0.3.

## Results

### Participants

**Figure 1:**
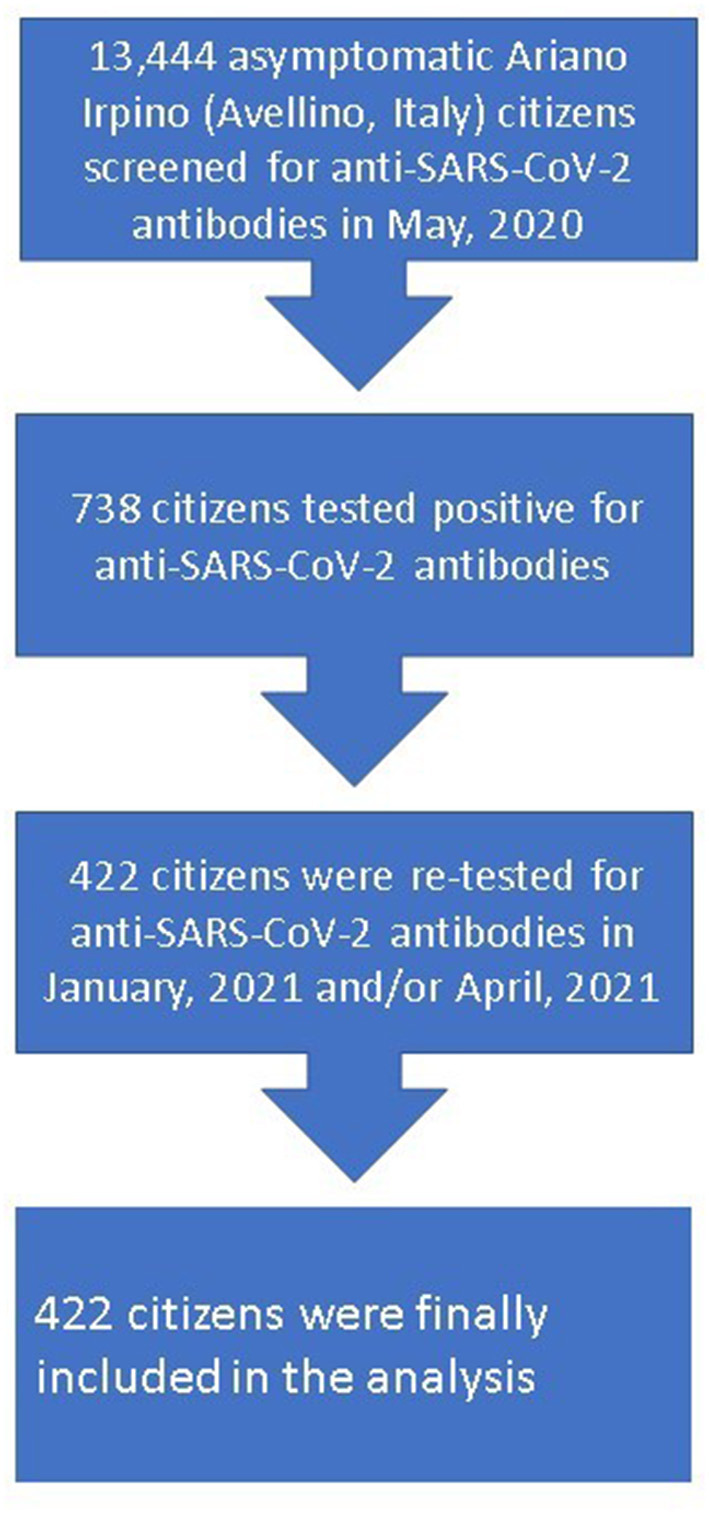
Flow-chart of study design.

SARS-CoV-2 serological findings regarding the 738 citizens who were enrolled in the Ariano Irpino SARS-CoV-2 Screening Program on 15/16th May, 2020 were reviewed for the purpose of this study. Among these, 422 citizens were finally included and retrospectively observed until April, 16 2021 (Figure 1). All included citizens had undergone a blood draw and a nasopharyngeal swab by trained personnel in Ariano Irpino on January 25 or January 26, 2021 and/or on April 27–28, that is 8.5 and 11.5 months after being found seropositive. Median (interquartile range) age of the study cohort was 46 years (29–59), and 47 (11.1%) participants were minors, while 217 (51.4%) participants were females. Naso-pharyngeal swabs were negative for all participants both in January and in April, 2021.

### Seroprevalence Findings

In the overall study cohort, seroprevalence varied widely according to the test employed. Presence of anti-N antibodies was reported in 95.7 and 93.7% of evaluable participants in January and April 2021, respectively, using the Elecsys® Anti-SARS-CoV-2 immunoassay, while it was reported only in 54.4 and 34.8% of evaluable participants in January and April 2021, respectively, using the BioPlex 2200 SARS-CoV-2 IgG kit (Biorad). Presence of anti-S1 antibodies (Biorad) was found in 87.0 and 85.5% of evaluable participants in January and April 2021, respectively, while anti-S2 antibodies (Biorad) were found in 41.7 and 43% of participants in January and April 2021, respectively, and anti-RBD (Biorad) antibodies were reported in 92.9% and 91.8 of evaluable participants in January and April 2021, respectively.

No significant differences were found in the overall population, in males vs. females and in adults vs. minors when we compared serological test results obtained in January vs. April, 2021, with the exception of anti-N (Biorad) seroprevalence, which was consistently lower in April vs. January, 2021 in the overall population and in all sub-groups (*p* < 0.05, z score test) (Table 1).

**Table 1:** Prevalence of specific serum antibodies in the overall population, in adults vs. minors and in males vs. females in January and April, 2021.

		**Overall population**		**Males**		**Females**		**Minors**		**Adults**	
		**Jan 21**	**April 21**	**Jan 21**	**April 21**	**Jan 21**	**April 21**	**Jan 21**	**April 21**	**Jan 21**	**April 21**
Presence of anti-N antibodies (Roche)	Positive/N	401/419	385/411	195/202	188/198	206/217	197/213	43/45	41/43	358/374	344/368
	% (95% CI)	95.7 (93.8–97.7)	93.7 (91.2–96)	96.5 (94–99.1)	95 (91.9–98)	94.9 (92–97.9)	92.5 (89–96)	95.6 (89.5–100)	95.4 (89.1–100)	95.7 (93.7–97.8)	93.5 (91–96)
Presence of anti-S1 antibodies (Biorad)	Positive/N	367/422	354/414	173/205	166/200	194/217	188/214	46/47	42/43	321/375	312/371
	% (95% CI)	87 (83.8–90.2)	85.5 (82.1–88.9)	84.4 (79.4–89.4)	83 (77.8–88.2)	89.4 (85.3–93.5)	87.9 (83.5–92.2)	97.9 (93.8–100)	97.7 (93.2–100)	85.6 (82.1–89.2)	84.1 (80.4–87.8)
Presence of anti-S2 antibodies (Biorad)	Positive/N	176/422	178/414	73/205	78/200	103/217	100/214	25/47	20/43	151/375	158/371
	% (95% CI)	41.7 (37–46.4)	43 (38.2–47.8)	35.6 (29.1–42.2)	39 (32.2–45.8)	47.5 (40.8–54.1)	46.7 (40–53.4)	53.2 (38.9–67.5)	46.5 (31.6–61.4)	40.3 (35.3–45.2)	42.6 (37.6–47.6)
Presence of anti-N (Biorad)	Positive/N	192/421	144/414	92/205	68/200	100/216	76/214	29/47	17/43	163/374	127/371
	% (95% CI)	54.4 (49.6–59.2)	34.8 (30.2–39.4)	44.9 (38.1–51.7)	34 (27.4–40.6)	46.3 (39.7–53)	35.5 (29.1–41.9)	61.7 (47.8–75.6)	39.5 (24.9–54.2)	43.6 (38.6–48.6)	34.2 (29.4–39.1)
Presence of anti-RBD antibodies (Biorad)	Positive/N	388/422	380/414	184/205	181/200	204/217	199/214	46/47	42/43	342/375	338/371
	% (95% CI)	92.9 (89.4–94.5)	91.8 (89.1–94.4)	89.8 (85.6–93.9)	90.5 (86.4–94.6)	94 (90.9–97.2)	93 (89.6–96.4)	97.9 (93.8–100)	97.7 (93.2–100)	91.2 (88.3–94.1)	91.1 (88.2–94)

*Qualitative assessment*.

Results of semi-quantitative analysis are shown in Table 2. The semi-quantitative nature of the analysis poses a major limitation to analyze differences, a titer >100 IU/L was reported more frequently for anti-S1 and anti-RBD antibodies (Biorad) compared to anti-S2 and anti-N antibodies.

**Table 2:** Prevalence of specific serum antibodies in the overall population, in adults vs. minors and in males vs. females.

		**Overall population**		**Males**		**Females**		**Minors**		**Adults**	
		**Jan 21**	**April 21**	**Jan 21**	**April 21**	**Jan 21**	**April 21**	**Jan 21**	**April 21**	**Jan 21**	**April 21**
**Relative frequencies of the positive subjects (95% CI)**											
<1 IU / ml	Anti-S1 antibodies (Biorad)	3.1% (1.4–4.7)	2.7% (1.1–4.2)	3.9% (1.3–6.6)	2.5% (0.3–4.7)	2.3% (0.3–4.3)	2.8% (0.6–5)	0% (–)	0% (–)	3.5% (1.6–5.3)	3.0% (1.2–4.7)
	Anti-S2 antibodies (Biorad)	4.0% (2.2–5.9)	3.9% (2–5.7)	4.9% (1.9–7.8)	3.5% (1–4.7)	3.2% (0.8–5.6)	4.2% (1.5–6.9)	0% (–)	2.3% (0–6.8)	4.5% (2.4–6.6)	4.0% (2–6.1)
	Anti-N (Biorad)	7.6% (5.1–10.1)	15% (11.8–18.7)	8.3% (4.5–12.1)	16% (10.5–20.5)	6.9% (3.6–10.3)	15% (10.2–19.7)	4.3% (0–10)	2.3% (0–6.8)	8.0% (5.3–10.8)	17% (12.9–20.5)
	Anti-RBD antibodies (Biorad)	3.1% (1.4–4.7)	2.7% (1.1–4.2)	3.9% (1.3–6.6)	2.5% (0.3–4.7)	2.3% (0.3–4.3)	2.8% (0.6–5)	0% (–)	0% (–)	3.5% (1.6–5.3)	3.0% (1.2–4.7)
**Relative frequencies of the positive subjects (95% CI) / Median [interquartile range]**											
1–100 IU / ml	Anti-S1 antibodies (Biorad)	59% (54.8–64.2) 24 [12–46.5]	54% (49.3–59) 20 (10–41)	63% (56.3–70) 23 (11–43)	54% (46.6–60.4) 17 [9–34.5]	56% (49.6–62.8) 28.5 [13.3–48]	55% (48–61.3) 25 [11–47]	40% (26.4–54.5) 56 [38–82]	35% (20.6–49.1) 48 [35.5–55]	62% (57–66.8) 22.5 [11–44.3]	56% (51.3–61.4) 19 [10–36]
	Anti-S2 antibodies (Biorad)	92% (89.4–94.5) 7 [4–15]	82% (78.4–85.8) 7 [3–13]	92% (87.9–95.5) 6 [3–14.3]	82% (76.1–86.9) 5 [5–12.5]	92% (88.6–95.8) 8.5 [5–16]	83% (77.6–87.8) 8 [4–14]	98% (93.8–100) 10 [5.3–14]	95% (89.1–100) 9 [6–13]	91% (88.3–94.1) 7 [3–16]	81% (75.6–84.6) 6 [3–14]
	Anti-N (Biorad)	88% (85–91.2) 9 [3–26.5]	83% (79.7–86.9) 6 [3–18]	88% (83.3–92.3) 9 [3.8–28]	83% (77.8–88.2) 6 [2–15]	88% (84.2–92.7) 8 [3–26]	84% (78.7–88.6) 5 [3–19]	89% (80.6–96.2) 14.5 [6–31]	95% (89.1–100) 6 [3–15]	88% (84.7–91.3) 8 [3–26]	82% (78–85.9) 5 [3–18]
	Anti-RBD antibodies (Biorad)	50% (45–54.5) 39.5 [21.3–65]	44% (39.7–49.2) 31 [17.8–55]	55% (48.3–61.9) 38 [20–65]	47% (40.1–53.9) 26.5 [16–50.8]	45% (38.1–51.3) 41 [23–65]	42% (35.4–48.7) 35.5 [20–56]	26% (13.1–38) 61 [31.5–90.3]	23% (10.6–35.9) 74 [43.3–94]	53% (47.7–57.9) 37.5 [21–64]	47% (41.8–52) 30 [17–51]
**Relative frequencies of the positive subjects (95% CI)**											
>100 IU / ml	Anti-S1 antibodies (Biorad)	37% (32.8–42.1)	43% (38.5–48)	33% (26.7–39.6)	44% (37.1–50.9)	41% (34.9–48)	43% (35.9–49.2)	60% (45.5–73.6)	65% (50.9–79.4)	35% (29.9–39.5)	41% (35.7–45.7)
	Anti-S2 antibodies (Biorad)	4.0% (2.2–5.9)	14% (10.7–17.4)	3.4% (0.9–5.9)	15% (10.1–20)	4.6% (1.8–7.4)	13% (8.6–17.6)	2.1% (0–6.3)	2.3% (0–6.8)	4.3% (2.2–6.3)	15% (11.7–19)
	Anti-N (Biorad)	4.3% (2.3–6.2)	1.4% (0.3–2.6)	3.9% (1.3–6.6)	1.5% (0–3.2)	4.6% (1.8–7.4)	1.4% (0–3)	6.4% (0–13.4)	2.3% (0–6.8)	4.0% (2–6)	1.3% (0.2–85.9)
	Anti-RBD antibodies (Biorad)	47% (42.4–51.9)	53% (48.1–57.7)	41% (34.2–47.7)	51% (43.6–57.4)	53% (46.4–59.6)	55% (48.5–61.8)	74% (62–86.9)	77% (64.1–89.4)	44% (38.7–48.8)	50% (45.1–55.2)

*Semi-quantitative assessment*.

Multiple logistic regression analysis used to explore associations between age, sex and seroprevalence showed that adults vs. minors had lower odds of having anti-S1 antibodies (Biorad) both in January and in April, 2021 (OR = 0.12; *p* = 0.03), while other statistically significant associations found in January, 2021 were not confirmed in April, 2021 (Table 3).

**Table 3:** Multiple logistic regression analysis.

		**Presence of anti-N antibodies (Roche)**		**Presence of anti-S1 antibodies (Biorad)**		**Presence of anti-S2 antibodies (Biorad)**		**Presence of anti-N (Biorad)**		**Presence of anti-RBD antibodies (Biorad)**	
		**OR (95%CI)**	***P*-value**	**OR (95%CI)**	***P*-value**	**OR (95%CI)**	***P*-value**	**OR (95%CI)**	***P*-value**	**OR (95%CI)**	***P*-value**
Age group (minors = ref)	Jan 2021	1.08 (0.17, 4.01)	0.917	0.12 (0.01, 0.57)	**0.039**	0.55 (0.29, 1.02)	0.058	0.47 (0.25, 0.88)	**0.019**	0.21 (0.01, 1.01)	0.127
	April 2021	0.74 (0.12, 2.63)	0.687	0.12 (0.01, 0.56)	**0.036**	0.82 (0.43, 1.56)	0.541	0.79 (0.42, 1.54)	0.481	0.23 (0.01, 1.12)	0.154
Sex (females = ref)	Jan 2021	1.49 (0.57, 4.12)	0.424	0.60 (0.34, 1.07)	0.087	0.59 (0.40, 0.88)	**0.009**	0.91 (0.62, 1.34)	0.633	0.53 (0.25, 1.08)	0.087
	April 2021	1.50 (0.67, 3.50)	0.333	0.63 (0.36, 1.10)	0.103	0.73 (0.49, 1.08)	0.112	0.93 (0.62, 1.40)	0.730	0.68 (0.33, 1.38)	0.288

*Age (adults vs. minors) and sex (males vs. females) were entered in the model to predict seropositivity assessed qualitatively. A p < 0.05 was considered statistically significant. The bold values indicate the p value minor of 0.05*.

## Discussion

**Figure 2:**
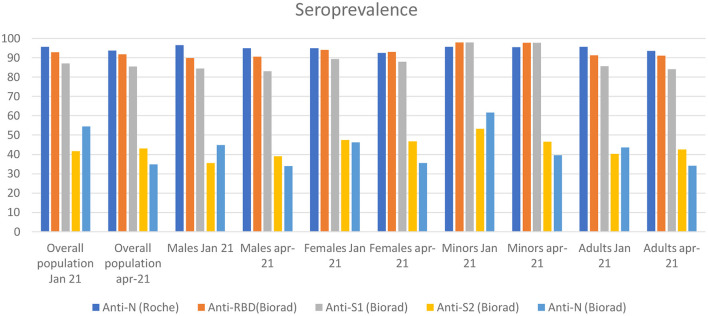
The figure shows the seroprevalence for each antibody isotype from May 2020 to April 2021 divided by overall population, males and females.

Approximately 90% of patients with symptomatic COVID-19 have anti-S and anti- RBD antibodies up to 8 months after onset of symptoms (12). Of note, humoral response may be less durable in asymptomatic individuals (6). In our longitudinal retrospective observational study, we assessed the presence of antibodies against anti-SARS-CoV-2 in a large cohort of never symptomatic, seropositive individuals enrolled in the Ariano Irpino SARS-CoV-2 Screening Campaign in May, 2020 (Figure 2). Importantly, >90% of individuals remained seropositive for anti-N antibodies (Roche) after an 11.5-month follow-up, without any evidence of re-infection during the observation period. Of note, all the citizens enrolled had tested negative for SARS-CoV-2 RNA at the time they were re-tested for anti SARS-CoV-2 antibodies in January and April, 2021. Our main finding is consistent with the results obtained in a longitudinal cross-sectional study (6) conducted in Wuhan since April 14–15, 2020 (baseline) to a time period between October 9 and December 5, 2020 (second follow-up). In the sub-group of 362 asymptomatic seropositive individuals with available baseline and second follow-up serological assessment, 329 (90.6%) were positive to anti-SARS-CoV-2 nucleocapsid protein IgG antibodies, but only 112 (40.0%) had neutralizing antibodies upon second follow-up. In our cohort, a similar proportion (41.7%) of individuals tested positive for anti- S2 antibodies. Anti-S2 antibodies may inhibit cell-cell membrane fusion (13, 14), we hypothesize that anti-S2 antibodies detected using the Biorad multiplex assay may contribute to the serum neutralizing capacity measured *in vitro* using microneutralization assays. This hypothesis may also be supported by the lower odds of having anti-S2 antibodies for males vs. females reported in January, 2021 in our study cohort, which was consistent with the numerically, albeit not statistically significant, lower prevalence of neutralizing antibodies reported by He et al. in males vs. females (35 vs. 41%) upon second follow-up. Assessment of anti-S2 and neutralizing antibodies is required to test this hypothesis. Our study results are also in line with a recent study in which immune memory was assessed for associations between magnitude of memory and COVID-19 disease severity in 188 patients with COVID-19. The authors observed that anti-S IgG titers were durable with a modest decline at 6–8 months post symptom onset. Notably, memory B cells specific for the spike protein or RBD were detected in almost all COVID-19 cases, with no apparent half-life at 5–8 months after infection (12). In our study, we showed higher levels of anti-S1 (median levels, 24 IU/ml) and anti-RBD (median levels, 39 IU/ml) antibodies compared to anti-S2 antibodies (7 IU/ ml), with a higher proportion of patients showing >100 IU/ml levels of anti-S1 and anti-RBD compared to anti-S2 antibodies. Further studies are required to interpret our findings and compare to those achieved by others. In this regard, it is worthy to mention the results reported in a cohort of 210 individuals followed-up for >6 months by Pradenas et al. (15), who concluded that the half-life of anti- RBD, anti-S2, and anti-nucleocapsid antibodies was 86, 108, and 59 days, respectively.

Other small serological studies have included only few asymptomatic subjects (16, 17), but have shown detectable antibody titers up to 6–9 months, which is consistent with the findings reported here. Conversely, in a retrospective study examining the results of >30,000 SARS-CoV-2 antibody tests performed between April and November 2020, ~50 % of seropositive participants at baseline with few or no symptoms or clinical disease had sero-reverted at 30 days (18). In our study, we found that only a small (1–2%) proportion of subjects had sero-reverted in April, 2021 compared to January, 2021. The small decrease in seroprevalence was consistent across anti -N (Roche), anti-S1 (Biorad) and anti-RBD antibodies. A paradoxical small increase in seroprevalence of anti-S2 (Biorad) antibodies was also found, for which we are unable to provide an explanation, along with large decrease in seroprevalence of anti-N antibodies (Biorad), which is consistent with a lower sensitivity of the Biorad assay compared to the Roche Elecysis assay (19).

Furthermore, we found evidence suggesting that children may have a more durable humoral response. In particular, adults had lower odds of having detectable anti- S1 antibody levels compared to minors (OR = 0.11, *p* < 0.01) at multivariate analysis and also had lower anti S1 titer levels in the subgroups of individuals with quantifiable titer levels (22 vs. 56 U/mL, respectively, *p* < 0.01). Lastly, we found that a higher proportion of individuals with anti-N antibodies measured by using Elecsys® Anti-SARS-CoV-2 (Roche) compared to Bio-Plex Multiplex SARS-CoV-2 Serology Assay (95.7 vs. 45.4%), which may reflect a higher sensitivity of the former method, as noted above.

Our study presents a number of limitations. First, we cannot exclude that enrolled individuals may have been re-exposed to SARS-CoV-2 at some time during the retrospective observation, although none of them has tested positive for SARS-CoV-2 at any time and all of them underwent RT-PCR testing in January, 2021. Second, the study cohort has not been assessed using multiplex Biorad assay for anti S1, S2, RBD and N IgG antibodies at baseline, which makes it more difficult to interpret the data obtained. Third, a proportion of individuals had > 100 IU / mL antibody levels and we were unable to provide the exact measurement of the antibody levels, as we did not perform additional dilutions of the samples. This represents a major limitation that prevents us from modeling antibody decay quantitatively. Fourth, we cannot exactly estimate time since first infection to serological assessment in the majority of the study cohort, although we presume that most of the individuals were infected not earlier than 2 months before May, 2020.

Despite these caveats, we believe that our study has the merit to be the largest ever conducted in a cohort of asymptomatic individuals who were tested for anti-nuclear capside antibodies after 11.5 months, at which time we proved that >90% showed serum antibodies. Also, we noted that sero-reversion is an infrequent (1–2%) event between 8.5 and 11.5 months after first antibody detection. Finally, we found that individuals who have anti-S2 antibodies represent a sub-group of those who have anti-S1 and anti-RBD antibodies, which may have clinical significance in immunoprotection, although, it needs to be further elucidated.

While our results have undoubtedly value from an epidemiological perspective, more studies are required to assess the duration of seropositivity after recovery from SARS-CoV-2 infection and its implications for mass vaccination programs.

## Data Availability Statement

The raw data supporting the conclusions of this article will be made available by the authors, without undue reservation.

## Ethics Statement

Ethical review and approval was not required for the study on human participants in accordance with the local legislation and institutional requirements. Written informed consent to participate in this study was provided by the participants' legal guardian/next of kin.

## Author Contributions

AC: conceptualization, methodology, data curation, investigation, writing—original draft, writing—review and editing, analysis, and project administration. CB: conceptualization, validation, writing—original draft, and writing—review and editing. DC and SD: writing review and editing and data curation. GLC, DS, and RS: writing review and editing and analysis. GR and DP: writing review and editing, data curation, and statistical analysis. MM and MT: conceptualization, writing review and editing, and investigation. LA, GB, AntP, and BP: conceptualization, methodology, writing review and editing, and investigation. AndP: Software, writing review and editing, and data curation. VF, MZ, MC, PA, and SR: conceptualization, methodology, and writing review and editing. SS: methodology and writing review and editing. PC: conceptualization, methodology, data curation, investigation, writing—review and editing, analysis, project administration, and supervision. All authors contributed to the article and approved the submitted version.

## Funding

This work was funded by POR FESR CAMPANIA 2014–2020 - O.S. 1.3 - AZIONE 1.3.1. DG 10. - PROGETTO Studio di Sorveglianza Sanitaria del virus SARS-CoV-2 responsabile della pandemia da COVID-19 nella popolazione ad alto rischio o esposta a contatto diretto con pazienti positivi - CUP C75I20000060002, approved by the Federico II Ethics Committee (n. 141/20) - GENCOVID - Studio di Sorveglianza Sanitaria del virus SARS-CoV-2 responsabile della pandemia da COVID-19 nella popolazione ad alto rischio o esposta a contatto diretto con pazienti positivi - Covid19 - GENCOVID. The authors have no other relevant affiliations or financial involvement with any organization or entity with a financial interest in or financial conflict with the subject matter or materials discussed in the manuscript apart from those disclosed. No writing assistance was utilized in the production of this manuscript.

## Conflict of Interest

The authors declare that the research was conducted in the absence of any commercial or financial relationships that could be construed as a potential conflict of interest.

## Publisher's Note

All claims expressed in this article are solely those of the authors and do not necessarily represent those of their affiliated organizations, or those of the publisher, the editors and the reviewers. Any product that may be evaluated in this article, or claim that may be made by its manufacturer, is not guaranteed or endorsed by the publisher.
